# Mouse Chow Composition Influences Immune Responses and Food Allergy Development in a Mouse Model

**DOI:** 10.3390/nu10111775

**Published:** 2018-11-16

**Authors:** Eleonore Weidmann, Nazanin Samadi, Martina Klems, Denise Heiden, Klara Seppova, Davide Ret, Eva Untersmayr

**Affiliations:** 1 Institute of Pathophysiology and Allergy Research, Center for Pathophysiology, Infectiology and Immunology, Medical University of Vienna, 1090 Vienna, Austria; eleonore.weidmann@gmx.at (E.W.); nazanin.samadi@meduniwien.ac.at (N.S.); martina.klems@meduniwien.ac.at (M.K.); denise.heiden@meduniwien.ac.at (D.H.); klara.seppova@gmail.com (K.S.); davide.ret@tuwien.ac.at (D.R.); 2 Division of Macromolecular Chemistry, Institute of Applied Synthetic Chemistry, Vienna University of Technology, 1060 Vienna, Austria

**Keywords:** food allergy, diet, mouse chow, experimental mouse model, oral immunizations, polyunsaturated fatty acids, vitamin D, soy, linseed oil

## Abstract

Our diet is known to substantially influence the immune response not only by support of mucosal barriers but also via direct impact on immune cells. Thus, it was of great interest to compare the immunological effect of two mouse chows with substantial differences regarding micro-, macronutrient, lipid and vitamin content on the food allergic response in our previously established mouse model. As the two mouse chows of interest, we used a soy containing feed with lower fatty acid (FA) amount (soy-containing feed) and compared it to a soy free mouse chow (soy-free feed) in an established protocol of oral immunizations with Ovalbumin (OVA) under gastric acid suppression. In the animals receiving soy-containing feed, OVA-specific IgE, IgG1, IgG2a antibody levels were significantly elevated and food allergy was evidenced by a drop of body temperature after oral immunizations. In contrast, mice on soy-free diet had significantly higher levels of IL-10 and were protected from food allergy development. In conclusion, soy-containing feed was auxiliary during sensitizations, while soy-free feed supported oral tolerance development and food allergy prevention.

## 1. Introduction

It is well established that composition of the diet plays a paramount role in establishing an adequate immune response in humans with impact on allergic diseases [1]. To give some examples, essential fatty acids (FA) such as polyunsaturated FAs are vital for an adequate body function but have to be incorporated with food, like linseed oil or fish due to the fact that mammals lack the double bonds introducing enzymes [2,3]. Having the capacity to regulate inflammation, polyunsaturated FA metabolites derive either from omega-6 (*n*-6) or omega-3 (*n*-3) FAs. While *n*-6 polyunsaturated FAs such as arachidonic acid, linoleic acid or γ-linoleic acid give rise to pro-inflammatory eicosanoid mediators such as prostaglandins, leukotrienes and lipoxins, *n*-3 polyunsaturated FAs such as α-linolenic acid, eicosapentaenoic acid (EPA) and docosahexaenoic acid (DHA) are described to be associated with immunomodulation [4,5,6,7]. To give an example, EPA, a metabolite of α-linolenic acid is a precursor of 17-Hydroxyeicosapentaenoic acid (series E resolvin precursor) [8] or 17,18-epoxyeicosatetraenoic acid, which was repeatedly reported to possess anti-inflammatory and anti-allergic properties [9,10]. Recent studies show that especially the ratio of *n*-3 to *n*-6 polyunsaturated FAs is of great importance with regards to allergy development or allergy prevention. A higher amount of *n*-3 versus a lower amount of *n*-6 polyunsaturated FAs seems to be the ideal ratio to ensure a balanced healthy immune response [4,11,12]. However, within the “Western” high fat diet the content of *n*-6 polyunsaturated FAs is much higher compared to *n*-3 polyunsaturated FAs. This type of diet was recently revealed to contribute to allergy and asthma development in a large meta-analysis [13].

Other essential dietary components influencing the immune response are vitamins. Taking vitamin D as an example, controversial findings regarding impact on allergy development have been published. Poole and colleagues studied the epigenetic, genetic and cellular modulations of vitamin D concluding that numerous mechanisms might contribute to the positive impact of Vitamin D on allergy prevention [14,15,16,17]. In contrast, high levels of vitamin D by extensive maternal supplementation during pregnancy, birth, lactation or due to supplementation in early childhood was reported to be associated with enhanced allergen sensitizations [18,19,20]. Thus, there seems to be an U-shaped association between 25-hydroxyvitamin D (the active component of vitamin D) and IgE levels with either too low or too high levels of vitamin D being associated with allergy susceptibility [14,21,22].

With increasing knowledge regarding the influence of dietary components such as lipids, vitamins and other micronutrients on the appearance of inflammatory processes and the potential subsequent allergic development, control of immune-active dietary components influencing disease outcome is also essential for experimental models investigating mechanisms of type 2 immune responses [23]. Thus, food associated immune changes might be a crucial bias impeding comparability and reproducibility of results generated from experimental models. When aiming at establishing an oral sensitization protocol mimicking disease in food allergic patients, it is essential to prevent pre-exposure to the food protein of interest or any potentially cross-reactive proteins, as allergy induction might be impaired due to prior oral tolerance induction [24]. However, adequate diet control was reported to be not only essential in the animals involved in the experiments but also in parental generations or while suckling [24,25]. Besides control of food allergen exposure, there is a long list of dietary factors that can possibly change allergen-specific immune responses. Thus, in our present study we aimed to analyse the impact of two regular mouse chows used in animal housing for experimental research with different nutritional composition to evaluate the influence on the immune response in a previously established model of food allergy [26].

## 2. Materials and Methods

### 2.1. Animals and Diet

32 female Balb/c mice (Core Unit for Biomedical Research, Division for Laboratory Animal Science and Genetics, Himberg, Austria) were randomly divided in 4 groups of 8 and were kept under conventional condition (12 h light/dark cycle at 22 °C). 2 out of the 4 groups (a and n) were fed with LasQCdiet^®^Rod16-A (Soest, Germany), composed of a higher amount of long-chain polyunsaturated FAs due to the added linseed oil and lack of soybean product (soy-free feed; Table 1). The other 2 groups (A and N) were fed with ssniff fortified V1534-300 (Soest, Germany) consisting of a lower percentage of long-chain polyunsaturated FAs and soybean products (soy-containing feed; Table 1). All groups had unlimited access to water and food. 

Animals were treated according to European Union guidelines of animal care and the protocol has been approved by the local Ethics committee of the Medical University of Vienna and by the Austrian Federal Ministry of Science and Research with the approval number BMWFW-66.009/0182-WF/V/3b/2017.

Our model allergen of interest was Ovalbumin (OVA; Albumin from chicken egg white, Sigma Aldrich, Vienna, Austria, #A5503). As a control the mice were kept naive. For all treatments OVA was freshly dissolved in water to gain requested concentrations.

### 2.2. Immunization with OVA

On the first day of an immunization cycle each mouse of groups a and A received an intravenous injection of 116 µg proton pump inhibitor (PPI, Omep 40 mg^®^, Hexal AG, Holzkirchen, Germany) diluted in 100 µL 0.9% sterile sodium chloride solution. On the second and third day the mice were treated with another intravenous injection followed by an intramuscular injection one hour later with the same solution. 15 min after the intramuscular injection each mouse was fed with 0.2 mg OVA in combination with 2 mg sucralfate (Sucralan^®^, Gerot-Lannach, Vienna, Austria) in 100 µL of distilled water. 2 Groups (a and A) were exposed to the OVA immunization, 2 groups (n and N) stayed naive as negative controls (Table 2). The immunizations took place on the first three days of a two-week cycle. After 6 rounds of immunization mice were orally challenged and sacrificed. 

### 2.3. Oral Challenge with OVA

All 32 mice were orally challenged with 2 mg OVA in 100 µL double distilled water per mouse to provoke a systemic allergic reaction before sacrifice. Temperature was measured before as well as 10 min, 30 min and 1 h after oral gavage. The temperature was measured either with a rectal thermometer (Thermalert TH-5, Physitemp, Clifton, NJ, USA) or with a wireless temperature reader (DAS-7007S, PLEXX, Elst, Netherlands).

### 2.4. Measurement of OVA-Specific Antibody Levels

Blood was collected by heart puncture on sacrifice day to perform an enzyme-linked immunosorbent assay (ELISA) for evaluation of OVA-specific IgE, IgG1 and IgG2a levels. Purified mouse IgE Isotype control (0.1 µg/mL, BD Biosciences, Franklin Lakes, NJ, USA) and purified mouse IgG1 and IgG2a (0.4 µg/mL, SouthernBiotech, Birmingham, AL, USA) in 0.1 M sodium carbonate buffer, pH 9.6 (coating buffer) were used to prepare serial dilutions as standard curves. 96-well plates (Thermo Fisher Scientific, Waltham, MA, USA) were coated with 10 µg/mL OVA antigen in coating buffer and stored overnight at 4 °C. Plates were blocked with 1% dry milk powder (DMP) dissolved in tris-buffered saline containing 0.05% Tween (TBS-T) for 2 h at room temperature (RT). After blocking, samples were diluted in 0.1% DMP/TBS-T (1:20 dilution for IgE, 1:200 dilution for IgG1 and IgG2a) and left for soaking overnight at 4 °C. On the third day purified Rat Anti-Mouse IgE, IgG1 and IgG2a (1:500 dilution for all antibodies, BD Pharmingen, Heidelberg, Germany) in 0.1% DMP/TBS-T were added and left for 2 h at RT for detection of the OVA bound antibodies. ECL^TM^ anti-rat IgE horseradish peroxidase linked with whole antibody from goat (1:1000, GE Healthcare, Little Chalfont, UK) followed as an enzyme substrate. 

For detection of total and OVA-specific IgA intestinal levels, intestines were harvested after sacrifice and rinsed with 2 mL of extraction buffer (complete Mini, Roche, Basel, Switzerland). The intestinal content was extracted (4 h at 4 °C), centrifuged (5 min at 10 621g-force) and supernatants were collected and stored at −20 °C until further use. For total IgA detection, plates were coated with rat anti-mouse IgA (2 µg/mL, 100 µL/well, BD Biosciences) diluted in TBS overnight at 4 °C. TBS-T with 1% bovine serum albumin (BSA) was used for blocking (2 h at RT). Samples were diluted (1:1000 in 0.1% BSA/TBS-T) and the standard curve was prepared with purified mouse IgA isotype control (1.6 µg/mL in 0.1% BSA/TBS-T, BD Biosciences). Samples and standards were added to the wells and incubated for 30 min at RT. As detection antibody, biotin anti-mouse IgA (2 µg/mL in 0.1% BSA/TBS-T, 2 h at RT, BD Biosciences) was used and streptavidin horseradish peroxidase (1:5000 in 0.1% BSA/TBS-T, 1 h at RT, Vector Laboratories, Burlingame, CA, USA) as an enzyme substrate. For detection of OVA-specific IgA antibodies, plates were coated with OVA in coating buffer (10 µg/mL) as well as purified mouse IgA isotype control (BD Biosciences) for the standard curve (serial dilution from 0.1 µg/mL) overnight at 4 °C. After blocking (2 h at RT), undiluted samples (100 µL/well) were added to incubate overnight at 4 °C. Biotin anti-mouse IgA (1:250 in 0.1% BSA/TBS-T, BD Biosciences) was used as a detection antibody (2 h, RT) and streptavidin horseradish peroxidase (1:5000 in 1% BSA/TBS-T, 1 h at RT, Vector Laboratories) as an enzyme substrate afterwards.

For all ELISAs, the plates were washed manually at least 3 times with TBS-T between incubation steps. Tetramethylbenzidine (BD OptEIA TMB Substrate Reagent Set; BD Bioscience) was used as substrate and reaction was stopped with 1.8 mol/L H_2_SO_4_ 15 min at the latest. Read-out was made in the range of 450–630 nm using Infinite M200 Microplate reader (Tecan, Männedorf, Switzerland).

### 2.5. Splenocyte Stimulation and Cytokine Read-Out 

During sacrifice, spleen was removed and further processed under sterile conditions. Pre-lysis medium (RPMI medium 1640 with 1% Penicillin-Streptomycin (PenStrep) and 1% Glutamine (gibco, Thermo Fisher Scientific)) was added, centrifuged and the supernatant was removed. In total 5 mL of lysis buffer (ACK Lysing Buffer, Lonza, Gampel, Switzerland) was added and lysis was stopped with 5 mL complete medium (RPMI medium 1640 with 1% PenStrep, 1% l-glutamine and 10% foetal bovine serum (FBS)) after 5 min. Splenocytes were washed 2 times with 5 mL of complete medium, filtered and kept on ice until further use. After cell counting and adjusting splenocyte concentrations to 4 × 10^6^ cells/mL, cells were incubated with 3 stimulants: medium as a negative control, Concanavalin A (ConA) as a positive control and OVA (all 5 µg/mL). After a 72 h incubation period, stimulated splenocytes were centrifuged, supernatants collected and stored at −20 °C for later detection of cytokines.

For cytokine detection ready-to-use ELISA kits were utilized. Mouse IL-4 ELISA Ready-Set-Go! by eBioscience was deployed for detecting Interleukin 4 (IL-4) level and Mouse IL-10 Uncoated ELISA Kit by Invitrogen by Thermo Fisher Scientific for Interleukin 10 (IL-10) level. Instructions included were followed to achieve results.

### 2.6. Statistic and Data Analysis

All data was statistically analysed with GraphPad Prism version 7.00 for Windows (GraphPad Software, La Jolla, California, USA) [27]. The samples were tested for Gaussian distribution by Kolmogorov-Smirnov normality test. Groups among themselves were compared with Kruskal-Wallis non-parametric test and Dunn’s multiple comparison test as a post-hoc test.

## 3. Results

### 3.1. Mouse Feed Influences Systemic and Local Intestinal Antibody Production

**Figure 1:**
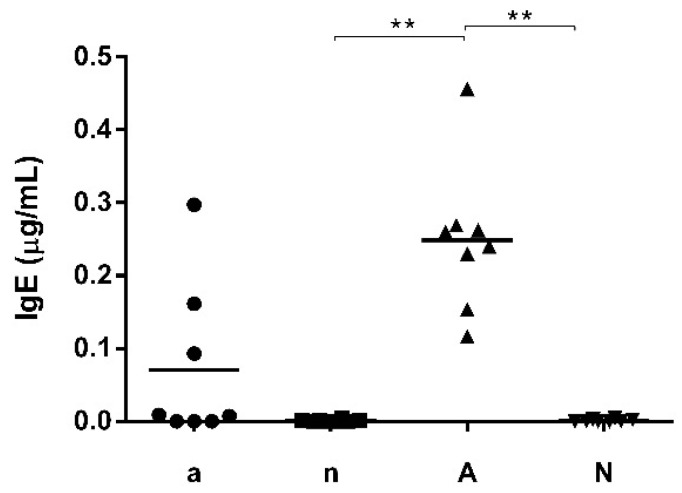
IgE levels in serum after immunization. Group a and n were fed with soy-free feed and group A and N with soy-containing feed. Groups A and a were immunized with 200 µg Ovalbumin (OVA) under gastric acid suppression. Mice of groups N and n were kept naive. Only in group A significantly higher IgE levels were measured than in the naive groups (n and N) (** *p* < 0.01).

**Figure 2:**
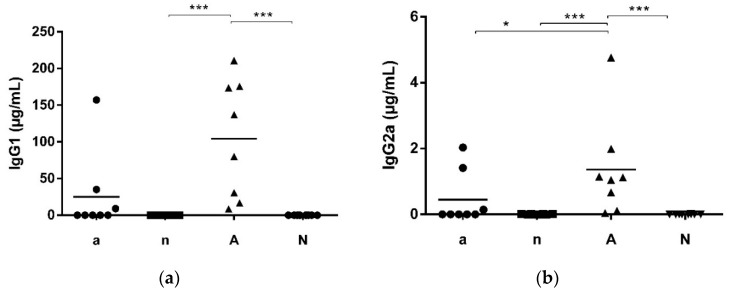
IgG1 and IgG2a serum levels after immunization. (**a**) IgG1 levels of group A were significantly increased in comparison to groups n and N; (**b**) IgG2a level of group A were significantly elevated compared to all other groups (* *p* < 0.05, *** *p* < 0.001).

Out of the 32 mice, two groups (each *n* = 8 mice) (a and n) were fed with soy-free feed whereas the two groups of A and N were fed with soy-containing feed. Groups a and A were immunized with OVA under gastric acid suppression for 6 times while groups n and N remained naive. Only in group A receiving soy-containing feed, IgE serum levels were significantly elevated in comparison to both naive groups (n and N; Figure 1). Comparable findings were observed when measuring IgG1 serum level. Group A showed significantly higher level than groups n and N (Figure 2a). For IgG2a serum levels, a significant difference was observed additionally between group A and a (Figure 2b). 

**Figure 3:**
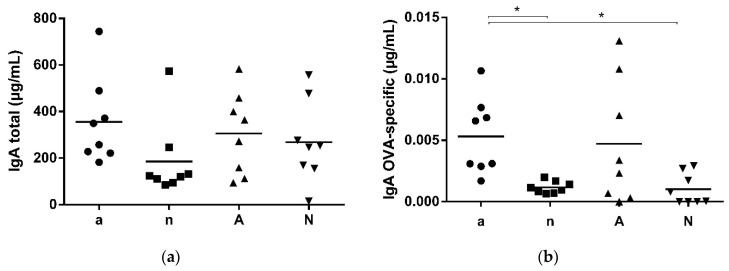
Total and OVA-specific IgA levels in intestinal lavages. After sacrifice, intestinal content was collected and screened for total and OVA-specific IgA levels. (**a**) No differences were revealed for the groups regarding total IgA levels; (**b**) Significantly higher OVA-specific IgA was detected in sera of group a, compared to the naive animals (groups n and N) (* *p* < 0.05).

To analyse the local immune response, we measured the total (Figure 3a) and OVA-specific (Figure 3b) IgA levels in intestinal lavages collected from the intestine directly after final experiments. Total IgA intestinal level (Figure 3a) were comparable between the four groups. OVA-specific IgA (Figure 3b) however, was significantly elevated in group a, compared to the naive mice of groups n and N.

### 3.2. Cytokine Production Reveal a Tolerogenic Milieu Based in Mouse Feed

**Figure 4:**
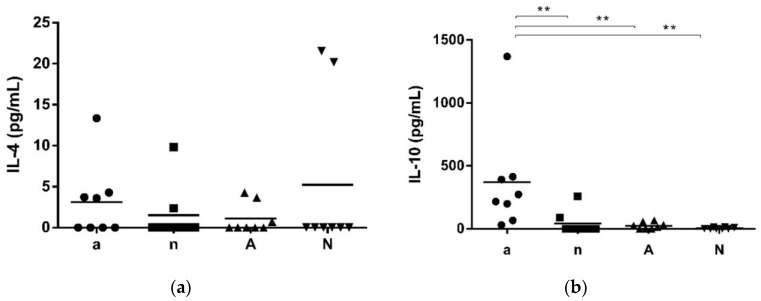
IL-4 and IL-10 levels in spleen cell supernatants after stimulation with OVA. (**a**) No differences regarding IL-4 levels were found between the groups. (**b**) IL-10 was significantly increased in group a, in comparison to the naive groups n and N (** *p* < 0.01).

Next, we evaluated the production of IL-4 and IL-10 in OVA stimulated spleen cells. All four groups showed no significant difference regarding the IL-4 concentration (Figure 4a). However, concentrations of IL-10 (Figure 4b) was significantly higher in group a, compared to all other groups. 

### 3.3. Temperature Changes after Oral OVA Challenge Indicate Food Allergy Development 

**Figure 5:**
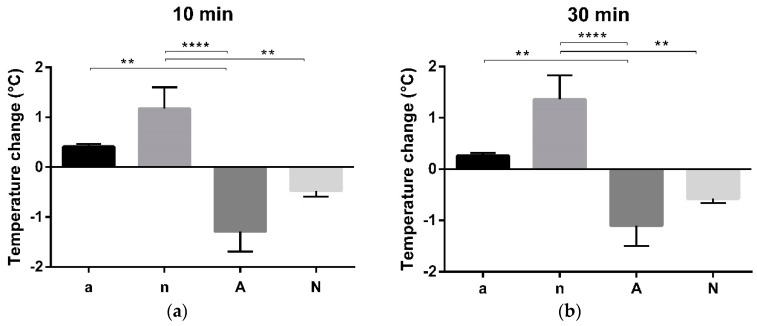
Temperature changes after oral OVA challenges. Temperature was measured before and 10 min, 30 min and 1 h after oral challenge. The drop of body temperature was calculated. (**a**) Group A showed a significant drop of temperature compared to groups a and n 10 min after oral challenge, while core body temperature of group N was also significantly lower compared to group n. (**b**) The same findings were observed 30 min after oral challenge. (** *p* < 0.01, **** *p* < 0.0001).

Before sacrifice, mice were orally challenged with 2 mg OVA and their core body temperature was measured. Mice of groups a and n revealed no changes in body temperature. In group A, a significant drop of temperature was measured 10 min (Figure 5a) and 30 min (Figure 5b) after oral challenge indicating food allergy. In the naive group N receiving soy-containing feed, a marginal decrease of body temperature was observed 10 min and 30 min after oral challenge (Figure 5a, b). 

## 4. Discussion

In our current study, we could detect a clear elevation of IgE, IgG1 and IgG2a level in serum of the immunized group fed with soy proteins and low FA amounts (soy-containing feed; group A) in comparison to the naive groups associated with a drop of core body temperature as a sign of anaphylaxis after oral challenge, indicating a clinically manifest IgE-mediated food allergy. Group a receiving the linseed oil based diet (soy-free feed) showed high levels of IL-10, which is known to be associated with regulatory T cell induction [28]. Furthermore, no elevated allergen specific IgE was observed, indicating a protection from food allergy development. In groups a and n, elevated levels of core body temperature have to be seen in context of enhanced activity of mice showing no clinical reaction upon oral allergen challenge. 

Composition of food substantially influences immune response and might contribute to development of food allergies. Due to westernization of the diet associated with elevated amounts of saturated and *n*-6 polyunsaturated FAs, lower levels of *n*-3 polyunsaturated FAs and an overuse of salt and refined sugar, negative effects on the immune system are observed [29]. With focus on the innate immune response, three major active components could be defined: low antioxidant intake, high fat intake and a chronic metabolic surplus associated with obesity and chronic inflammation [30]. Representing a risk factor for airway inflammation, high fat intake was discussed to enhance development of asthma via interference with the immune system [31,32]. A recent systematic review even concluded that fast food is associated with asthma and wheeze and other allergic diseases in a dose-response correlation [13].

Thus, for the prevention of food allergy development a healthy diet is of paramount importance and even influences the immune response before birth as the maternal diet provides the basis for developing the innate immune response in infants [33,34]. 

Without any doubt there are several dietary components of major importance with regards to immunity. Trace elements from dietary sources like iron are a direct target in the body’s defence against infection, as they are essential nutrients for pathogens. Recent studies concluded that also manganese and zinc are vital for bacterial survival [35]. By sequestration of zinc and manganese, bacteria are starved and dietary manganese levels were suggested to substantially influence intestinal microbiota composition with direct impact on the immune response [23,36]. Of interest, in our study manganese levels in the allergy protective soy-free feed were nearly double the amount as in the soy-containing feed. With regards to the influence of microbial composition on food allergy, our group has previously reported that protection from food allergy development is associated with the presence of distinct bacterial strains in faecal samples [37].

Another major difference of the two mouse chows was FA levels. A higher amount of *n*-6 polyunsaturated FAs than *n*-3 polyunsaturated FAs was proven to support allergy development in Balb/c mice [12] and is, thus, in line with our findings regarding the allergy development in group A. A higher ratio of *n*-6 to *n*-3 polyunsaturated FAs in human pregnancy was associated with an increased risk of allergic rhinitis in infants by the age of five years [38]. Increasing *n*-3 polyunsaturated FAs cannot eliminate or decrease the negative impact of westernized diet [39], although the anti-inflammatory metabolites of *n*-3 polyunsaturated FAs DHA and EPA can oppose the actions of *n*-6 polyunsaturated FAs especially concerning eicosanoid synthesis [4,40]. The ratio of *n*-6 to *n*-3 polyunsaturated FAs therefore plays an important role in balancing the immune response. As a healthy ratio, 4:1 *n*-6 to *n*-3 polyunsaturated FAs is suggested, while in western diet a ratio of 15–17:1 is detected [4,11,12]. The ratio of *n*-6 polyunsaturated FAs to *n*-3 polyunsaturated FAs in soy-containing feed was 8:1, while it is 3:1 in soy-free feed. 

Another difference in chow composition concerns vitamin D levels. In our study higher vitamin D levels were positively associated with protection from food allergy development. An U-shaped association between 25-hydroxyvitamin D and IgE level has been suggested [14,21,22]. Vitamin D has an essential impact on the immune system and allergy development via many mechanistic pathways [41]. Despite the fact that differences regarding vitamin D levels in the two mouse chows were small, it is known that also limited changes of dietary vitamin D levels impact on serum levels in rodents [42]. Moreover, in both chows vitamin D content is above national research council recommendations for rodents requirement [43]. Thus, due to the complex nutrient composition of both chows with high calcium levels an additional impact of vitamin D on the immune response cannot be excluded in this study. The observed elevated IL-10 levels could be ascribed to chow differences, as both vitamin D and *n*-3 polyunsaturated FAs are proven to have a positive impact on regulatory T cell induction [44,45].

Another main difference in mouse feed composition was the presence of soy proteins in the chow supporting allergy development. This is in contrast to findings from Chang and colleagues reporting that elimination of soy from the rodents feed resulted in sensitization to peanut [46]. Phytoestrogens or more specific isoflavones like daidzein and genistein in soy are well known to possess anti-inflammatory, antioxidative and chemo-preventive properties [47]. Consumed through the diet, they can suppress allergic sensitization in humans and supplementation could prevent a food allergy development [48]. However, in our study we observed the opposing effect as only mice receiving soy-containing feed were successfully sensitized to OVA. This might be due to mast cell activating function of oestrogens [49] which should be addressed in future research. 

One limitation in the current study is the fact that we did not measure plasma levels of the different immune modulating nutrients. However, due to the complexity of interactions, we expect that the observed impact on the immune response is due to an interplay of different components present in the mouse chow.

## 5. Conclusions

We found a significant correlation of different mouse chow composition pattern with allergy development in our experimental food allergy model. As has been underlined recently [50], diet composition and dietary control is of high relevance for rodent models and ignorance of dietary impact in experimental models might result in false conclusions. Moreover, our study supports the concept of allergy prevention via dietary components, which warrants extensive further research.

## Figures and Tables

**Table 1:** Dietary composition of different mouse chow according to manufacturers.

Soy-Containing Feed (Ssniff Rat/Mouse Maintenance Fortified V1534-300)	Soy-Free Feed (Lasvendi LasQCdiet Rod16-A)
Dietary Ingredients (in Descending Amount)	Dietary Ingredients (in Descending Amount)
Wheat soybean products barley minerals oat hulls molasses vitamins & trace elements L-lysine HCl DL-methionine	Wheat corn gluten oats corn barley linseed oil molasses brewer’s yeast minerals vitamins/trace elements-mix
**Energy per kg**	**Energy per kg**
Gross Energy (GE) 16.2 MJ Metabolizable Energy (ME) 13.5 MJ	Gross Energy (GE) 15.9 MJ Metabolizable Energy (ME) 13.1 MJ calc. formula §14 FMVO
**Minerals [%]**	**Minerals [%]**
Calcium 1.0 Phosphorus 0.7 Sodium 0.24 Magnesium 0.22 Potassium 0.92 Ca/P = 1.43: 1	Calcium 1.0 Phosphorus 0.65 Sodium 0.3 Magnesium 0.25
**Amino acids [%]**	**Amino acids [%]**
Arginine 1.19 Cysteine 0.35 Histidine 0.49 Isoleucine 0.79 Leucine 1.39 Lysine 1.1 Methionine 0.38 Phenylalanine 0.89 Threonine 0.72 Tryptophan 0.25 Alanine 0.87 Aspartic acid 1.84 Glutamic acid 4.22 Glycine 0.89 Proline 1.31 Serine 1.01 Valine 0.92 Met+Cys 0.73 Phe+Tyr 1.5	Arginine 0.8 Cysteine 0.35 Histidine 0.4 Isoleucine 0.65 Leucine 1.7 Lysine 0.9 Methionine 0.45 Phenylalanine 0.85 Threonine 0.6 Tryptophan 0.2 Tyrosine 0.6
**Vitamins per kg**	**Vitamins per kg**
Vitamin A 25,000 IU Vitamin D_3_ 1500 IU Vitamin E 135 mg Vitamin K (as MNB) 20 mg Thiamine (B1) 86 mg Riboflavin (B2) 32 mg Pyridoxine (B6) 31 mg Cobalamin (B12) 150 μg Biotin 710 μg Choline 1370 mg Folate 10 mg Pantothenate 59 mg Niacin 153 mg	Vitamin A 25,000 IU Vitamin D_3_ 1800 IU Vitamin E 120 mg Vitamin K 80 mg Thiamine (B1) 100 mg Riboflavin (B2) 30 mg Pyridoxine (B6) 25 mg Cobalamin (B12) 120 mg Biotin 400 µg Choline 1500 mg Folate 6 mg Pantothenate 35 mg Niacin 80 mg
**Trace Elements per kg**	**Trace Elements per kg**
Copper 15 mg Iodine 2.1 mg Iron 186 mg Manganese 68 mg Selenium 0.3 mg Zinc 91 mg	Copper 15 mg Iodine 4 mg Iron 200 mg Manganese 120 mg Selenium 0.2 mg Zinc 75 mg Cobalt 1.5 mg
**Fatty Acids [%]**	**Fatty Acids [%]**
C 14:0 0.01 C 16:0 0.45 C 18:0 0.09 C 20:0 0.01 C 16:1 0.01 C 18:1 0.62 C 18:2 1.76 C 18:3 0.23	C 16:0 0.5 C 18:0 0.2 C 20:0 0.01 C 18:1 0.9 C 18:2 1.9 C 18:3 0.75

^1^ Physiological fuel value (Atwater), corresponds to 3230 kcal/kg. Abbreviations: L = levo; HCl = hydrochloride; DL = dextrolevo; FMVO = Futtermittelverordnung (animal feed regulations).

**Table 2:** General overview.

Group	Sensitization	Oral Challenge	Number of Mice	Mouse Chow
a	200 µg OVA oral + acid suppression	2 mg/mL OVA oral	8	soy-free feed
n	naive	2 mg/mL OVA oral	8	soy-free feed
A	200 µg OVA oral + acid suppression	2 mg/mL OVA oral	8	soy-containing feed
N	naive	2 mg/mL OVA oral	8	soy-containing feed

Abbreviations: OVA = Ovalbumin.
